# Supplementary material for: Deciphering the functional and structural complexity of the Solar Lake flat mat microbial benthic communities
Source: mSystems. 2024 May 10;9(6):e00095-24. doi: 10.1128/msystems.00095-24 (PMC11237645; doi:10.1128/msystems.00095-24)
Supplement: Supplemental material — s and methods and Figures S1 to S6. [file msystems.00095-24-s0001.docx]

Supplementary file

**Supplementary material and method**

MAGs phylum level relative abundance Beta diversity was inferred using Bray–Curtis dissimilarities as calculated by the vegan R package 2.6-4 (1). Differences between shallow-water and exposed mats were visualized by principal coordinates analysis (PCoA).

The Compute Peak-to-Trough ratio (CoPTR, version 1.1.6) tool was used to calculate the growth dynamics of microbial taxa represented by 40 unique high-quality MAGs (< 95% average nucleotide identity (ANI), Completeness > 90%, and contamination <5%) (2).

The codes and scripts used in analyzing the data presented in the manuscripts are below:

**Sequence QC (paired-end)**

#The following step was applied on the 12 samples (3 replicas from the following samples : N-SO_Shallow-water_, N-SO_exposed_, E-SO_Shallow-water,_ and E-SO_Exposed_)

fastp -i raw-read-1.fastq.gz -I raw-read-2.fastq.gz -o QC–1.fq.gz -O QC-2.fq.gz

**Protein-based taxonomy based on reads**

kaiju -t nodes.dmp -f / kaiju_db_nr.fmi -i QC-fq -j QC-SO11-2.fq -o Outputfiles.out -z 80

#-r was applied on Phylum and Family level

# Output_files_from_previous_step = SO11-kaiju.out SO12-kaiju.out SO13-kaiju.out SO21-kaiju.out SO22-kaiju.out SO23-kaiju.out SO31-kaiju.out SO32-kaiju.out SO33-kaiju.out SO41-kaiju.out SO42-kaiju.out SO43-kaiju.out

kaiju2table -t nodes.dmp -n names.dmp -r family -o “Outputfile.tsv” “ Output_files_from_previous_step” -u -p

**Assembly**

megahit -1 Relplica1, 2, 3 (F reads) -2 Relplica1, 2, 3 (R reads) -o “MH-assembly-sample-name” --presets meta-large -m 0.85 -t 30

**Mapping**

bbmap.sh minid=0.9 ref=Assembly.contigs.fa in=reads_forward in2=reads_reverse threads=24 nodisk out=reads.vs.contigs.90.minid.bam bs=bs.sh covstats=reads.vscontigs.90.minid.covstats.txt scafstats=reads.vs.contigs.90.minid.scafstats.txt ; sh bs.sh

**Binning**

runMetaBat.sh -m 1500 -t 48 Assembly.contigs.fa replicate1.reads.vs.contigs_sorted.bam replicate2.reads.vs.contigs_sorted.bam replicate3.reads.vs.contigs_sorted.bam

**Filtering assemblies (reformat-bbmap)**

reformat.sh in=final.contigs.fa out=filtered-assemblyname.fasta minlength=500

**Functional annotation using DRAM**

DRAM.py annotate -i "input_bins/*.fa" -o output_dir_DRAM --use_uniref --min_contig_size 500 --threads 16

DRAM.py distill -i output_dir_DRAM/annotations.tsv -o output_dir_DRAM/genome_summaries --trna_path output_dir_DRAM/trnas.tsv --rrna_path output_dir_DRAM/rrnas.tsv

**GTDB-tk**

gtdbtk classify_wf --mash_db /path/to/gtdbtk-2.3.2/db/mash/ --cpus 16 --pplacer_cpus 16 -x fa --genome_dir input_bins --out_dir input_bins_gtdbtk

**Cazyme annotation**

#Screen the protein file of each MAG using HMM and diamond using dbcan webserver. Manually compile the results of the two sources. The compiled file should have column 1 for gene ID, column 2 for EC number if found and column 3 should have the Cazy family. Using the following script count the number of unique Cazy families in each bin.

awk -F, '{c[$3]++} END{for (i in c) print i, c[i]}' S-SO2-bin107-Cazy.txt > S-SO2-bin107-Cazy-sum.txt

**DRAM orf Coverage:**

The following script was used to calculate the orf coverage that was used for CPM calculations: <https://github.com/aelbehery/DRAM_extract_orf_cov>

References:

1. Dixon P. VEGAN, a package of R functions for community ecology. *J. Veg. Sci.* 2003;14(6):927-30.

2. Joseph TA, Chlenski P, Litman A, Korem T, Pe’er I. Accurate and robust inference of microbial growth dynamics from metagenomic sequencing reveals personalized growth rates. *Genome Res*. 2022;32(3):558-68.

**Supplementary Figures**


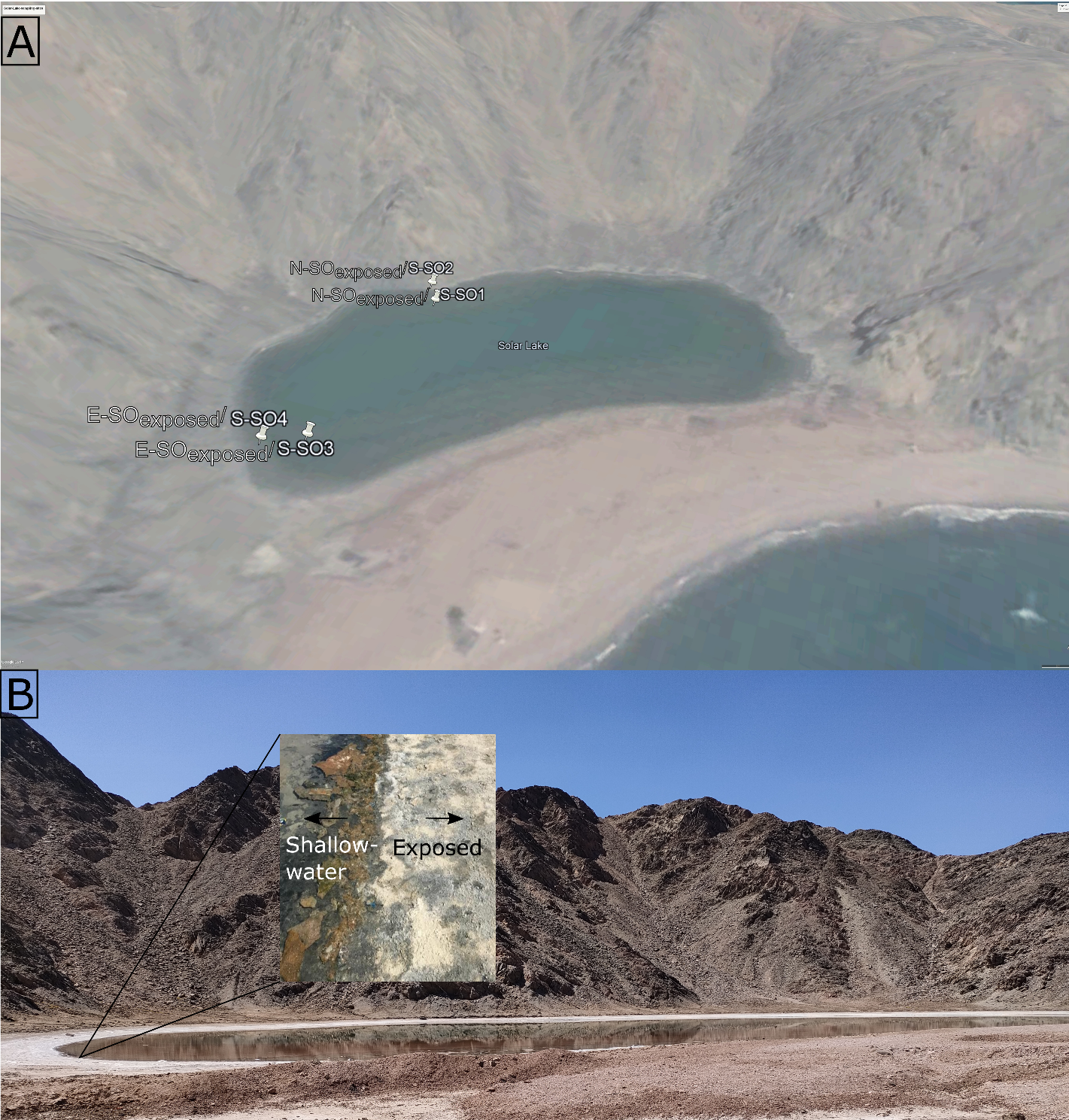


**Figure S1. Solar Samples sampling sites. (A)** The figure shows the Solar Lake sampling sites’ GPS coordinates imposed on a historical google earth image taken on 23/3/2021. **N-SO_shallow-water_ (S-SO1)** GPS coordinates: N 29.422549, E 34.829606, **N-SO_exposed_ (S-SO2)** GPS coordinates: N 29.4226111, E 34.82956, **E-SO_shallow-water_** **(S-SO3)** GPS coordinates: N 29.421962, E 34.829594, **E-SO_exposed_ (S-SO4)** GPS coordinates: N 29.4219167, E 34.8295. **(B)** An actual figure taken on the sampling day with a zoom-in on the microbial mate indicates where the Shallow-water and exposed flat microbial mat samples were collected.


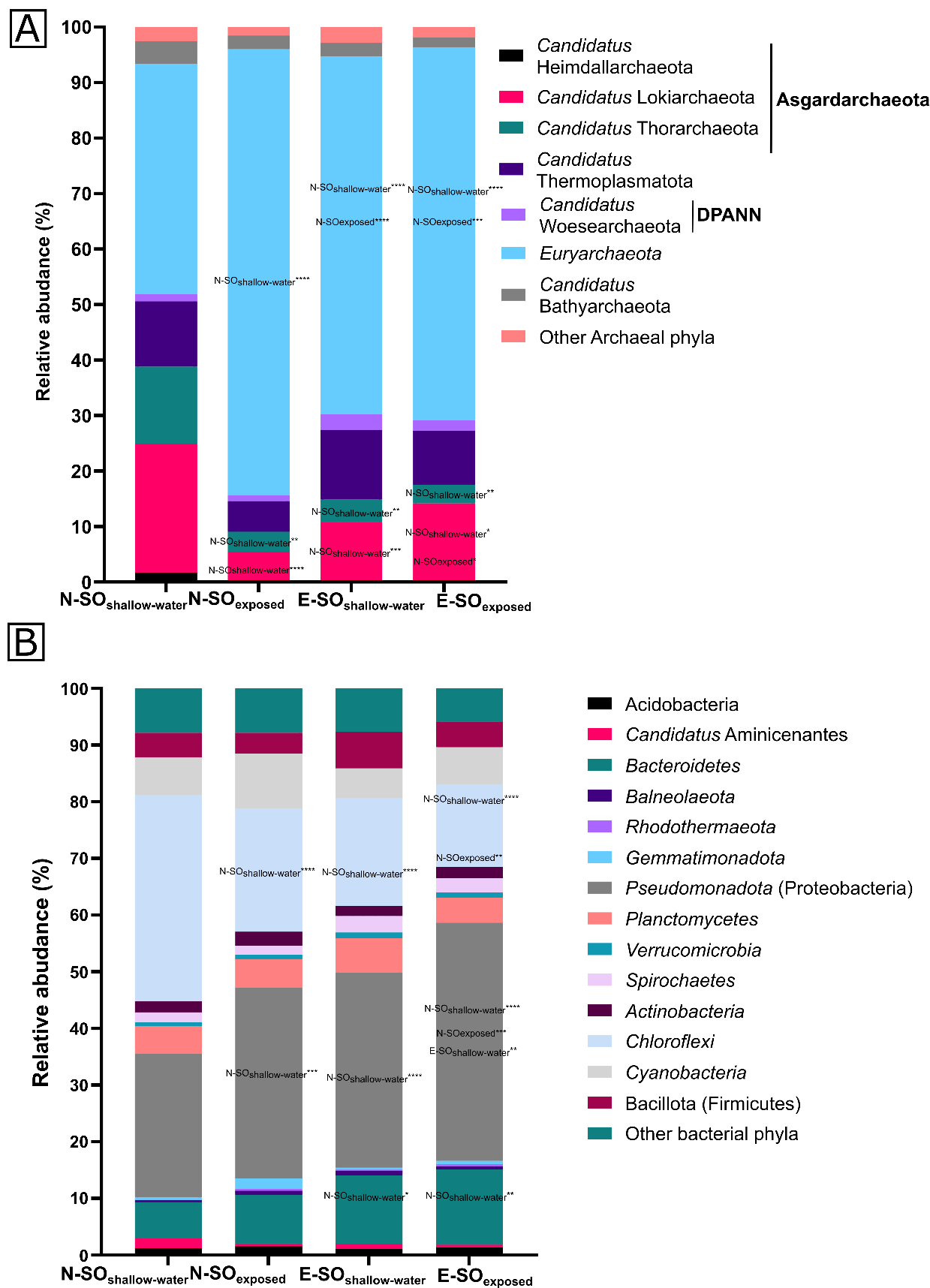


**Figure S2. Phylum-level classification of the Solar-lake flat mats metagenomic raw reads.** Community structure on phylum level. The graphs show the Kaiju computed relative abundance of (A) archaeal phyla within the archaeal community, and (B) Bacterial phyla within the bacterial community. It is to be noted, that around ~ 67% of the raw reads were unclassified, ~1.6 belonged to Eukaryotic taxa, and ~ 0.01% belonged to viruses. Pairwise comparisons p-values between the 4 different summer samples are shown (p-value ≤ 0.1 =*,≤ 0.01=**,≤ 0.001 =***, ≤ 0.0001 = ****).

**Figure S3. Carbohydrate degradation genetic potential (Carbohydrate active enzyme-Cazyme) in the Solar Lake flat mat assemblies.** The bar graph shows the CPM of CAZyme families genes involved in the degradation of amorphous cellulose, arabinan, mixed linkage glucan, polyphenolics, xyloglucans and sulf-polysaccharides in each Solar-lake assembly. Amorphous cellulose = GH16 + GH5 Cazyme families, polyphenolics = AA1 Cazyme family, sul-polysaccharide = GH16 + PL12 + PL6 + PL8 + PL33 Cazyme families, arabinan = GH43 Cazyme family, xyoglucan = GH16 + GH5 + GH43 Cazyme families, mixed linkage glucan = GH16 + GH30 + GH5 Cazyme families. The P-values of the pairwise comparisons between the Solar-lake assemblies Cazyme families CPM are shown on top of each comparison (P-value ≤ 0.1 =*,≤ 0.01=**,≤ 0.001 =***, ≤ 0.0001 = ****). S-SO1 and S-SO3 are shallow-water mats, while S-SO2 and S-SO4 are exposed mats.


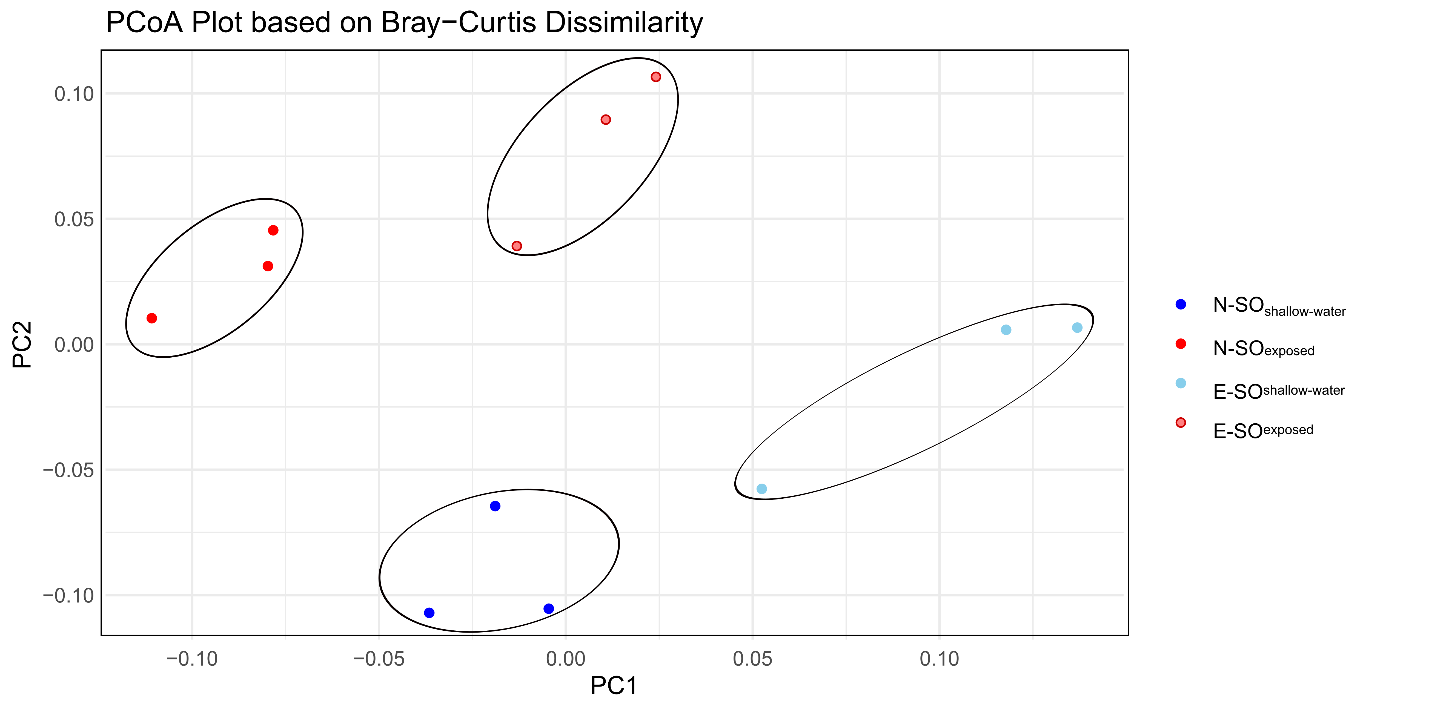


**Figure S4. Beta-diversity of the Solar Lake shallow water and exposed flat mat samples.** The PCoA plot is based on the BaryCurtis dissimilatory matrix applied on the phylum-level MAGs relative abundance. Blue color represents the shallow-water flat mats, and red color represents the exposed flat mats.


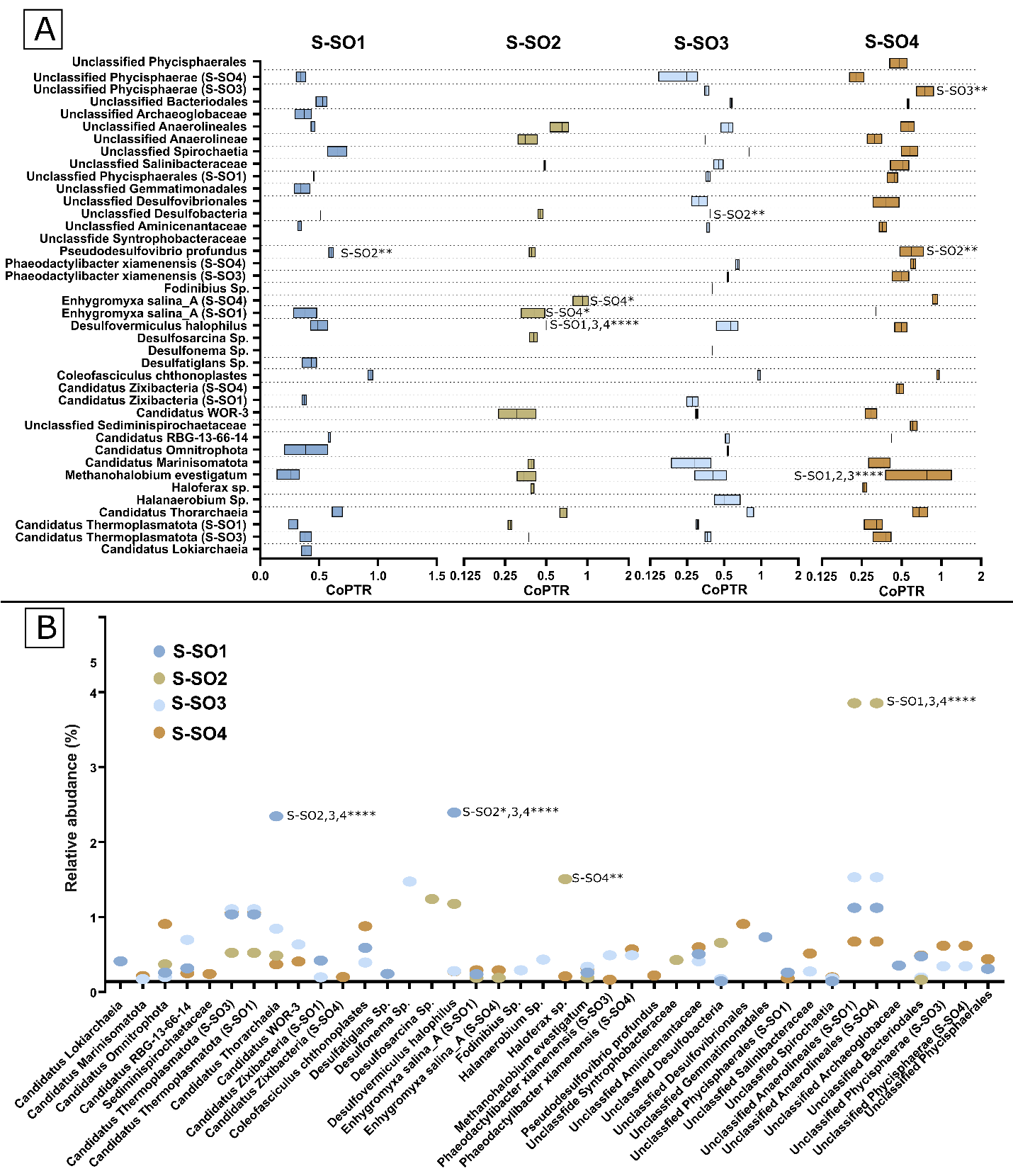


**E-SO_exposed_**

**E-SO_shallow-water_**

**N-SO_exposed_**

**N-SO_shallow-water_**

**Figure S5:** **Replication rate profiles (PTR) of high quality MAGs from the Solar Lake shallow-water and exposed mats.** The graph shows the replication rates (PTR) values for bacterial and archaeal species (mean value is indicated by the line). Statistical significance difference between samples was evaluated with Turkeys multiple test comparison, where: *= P≤0.05, ** = P≤0.01, ***= P ≤0.001, and **** = P≤0.0001. MAGs shown are of high-quality (70 %≥ complete and 5% ≤ contaminated) and are unique based on average nucleotide identity (ANI) <95%.


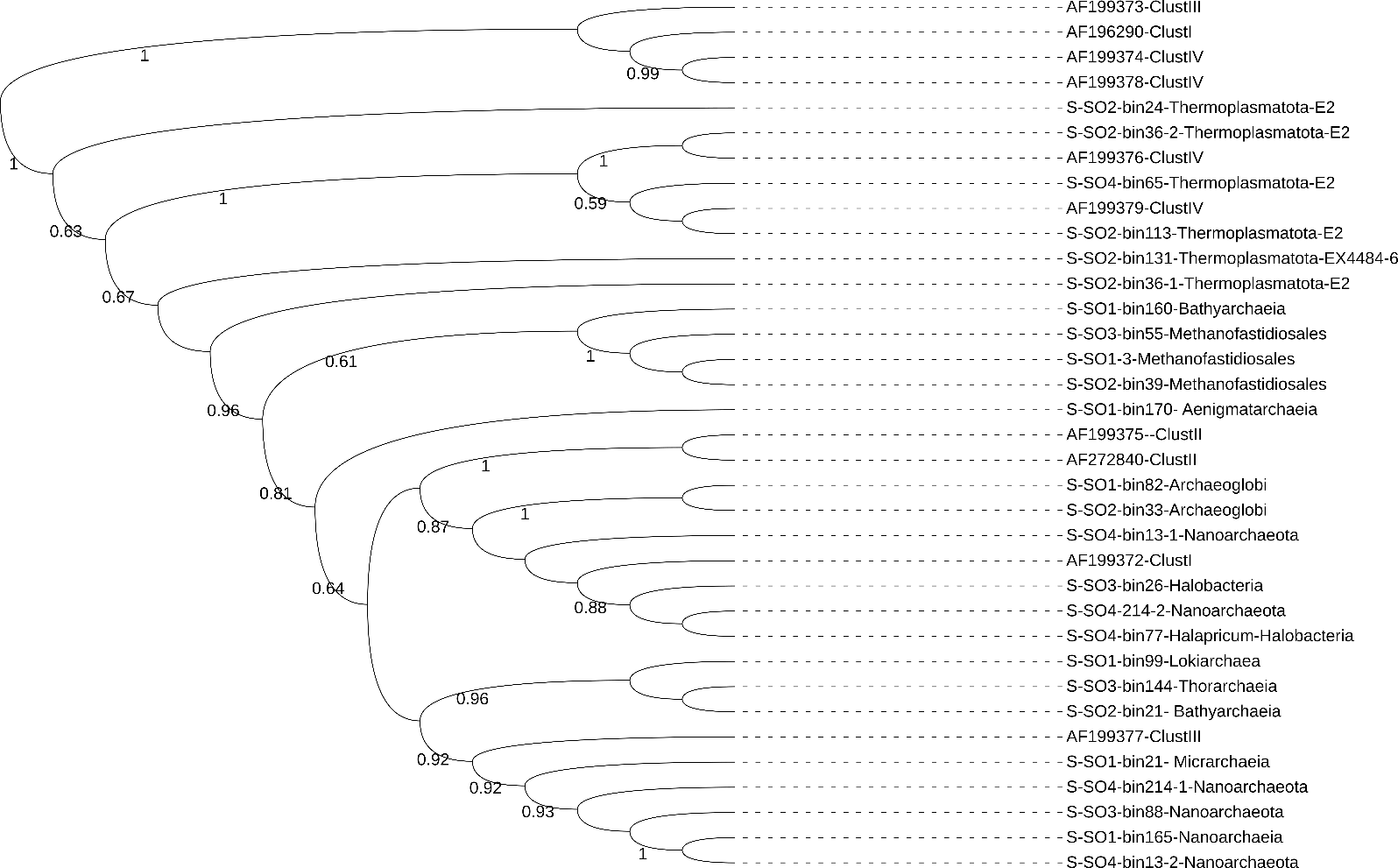


**Figure S6:** **Phylogenetic tree of 16S rRNA genes retrieved from this study Archaeal MAGs and the clone library of Cytryn *et al*, 2000.** The tree was generated using the maximum likelihood algorithm. Node with support > 50 % (0.5) are shown on three.
